# Brief cognitive screening instruments for early detection of Alzheimer’s disease: a systematic review

**DOI:** 10.1186/s13195-019-0474-3

**Published:** 2019-02-28

**Authors:** Ellen Elisa De Roeck, Peter Paul De Deyn, Eva Dierckx, Sebastiaan Engelborghs

**Affiliations:** 1 Department of Clinical and Lifespan Psychology, Vrije Universiteit Brussel, Brussels, Belgium; 2 Laboratory of Neurochemistry and Behavior, Institute Born-Bunge, University of Antwerp, Antwerp, Belgium; 3 Department of Neurology and Center for Neurosciences, UZ Brussel and Vrije Universiteit Brussel, Brussels, Belgium

**Keywords:** Cognitive screening, Alzheimer’s disease, Mild cognitive impairment, MCI, Pencil and paper tests, Computer tests, MMSE, MoCA

## Abstract

**Objectives:**

The objective of this systematic review was (1) to give an overview of the available short screening instruments for the early detection of Alzheimer’s disease (AD) and (2) to review the psychometric properties of these instruments.

**Methods:**

First, a systematic search of titles and abstracts of PubMed and Web of Science was conducted between February and July 2015 and updated in April 2016 and May 2018. Only papers written in English or Dutch were considered. All full-text papers about cognitive screening instruments for the early detection of AD were included, resulting in the identification of 38 pencil and paper tests and 12 computer tests. In a second step, the psychometric quality of these instruments was evaluated. Therefore, the same databases were searched again to identify papers that described the psychometric properties of the instruments meanwhile applying diagnostic criteria for the diagnostic groups included.

**Results:**

Out of 1454 papers, 96 clearly discussed the psychometric properties of the instruments. Eighty-nine papers discussed pencil and paper tests of which 80 were validated in a memory clinic setting. Based on the number of studies (31 articles) and the sensitivity (84%) and specificity (74%) values, the Montreal Cognitive Assessment (MoCA) seems to be a promising (pencil and paper) screening test for memory clinic testing as well as for population screening. Regarding computer tests, validation studies were only available for 7 out of 12 tests.

**Conclusions:**

A large number of screening tests for AD are available. However, most tests are only validated in a memory clinic setting and description of the psychometric properties of the instruments is limited. Especially, computer tests require further research. The MoCA is a promising instrument, but the specificity to detect early AD is rather low.

**Electronic supplementary material:**

The online version of this article (10.1186/s13195-019-0474-3) contains supplementary material, which is available to authorized users.

## Background

The aging population in Europe has been growing rapidly. According to the United Nations in 2015, 17.6% of the European population was older than 65 years. This will probably increase to 23.1% in 2030. It is therefore not surprising that more and more people (will) develop age-related diseases such as Alzheimer’s disease (AD). The process of AD pathology can be described as a continuum with a long preclinical phase without clinical symptoms, an early clinical phase in which mild clinical symptoms (mild cognitive impairment (MCI) or prodromal AD) are present, and a dementia phase [1–3]. For an effective intervention (including counseling, psycho-education, cognitive training, medication), early detection of the disease is important [4]. The same holds true for clinical trials with potential disease-modifying drugs for AD that increasingly focus on the earliest stages of the disease.

Cognitive screening instruments are cheap, fast, and non-invasive tools to identify adults at risk to have symptomatic AD. At present, the most used cognitive screening instrument for the detection of AD is the Mini-Mental State Examination (MMSE [5]). However, Mitchell concluded after a meta-analysis that the MMSE has a very limited ability to differentiate between MCI and healthy controls [6]. In agreement with Mitchell’s results, some reviews [7, 8] suggest to replace the MMSE by more performant alternatives. There are already a wide variety of alternative screening instruments in circulation. However, it is not yet clear which tests are sensitive and specific enough to detect AD in an early phase. Moreover, not every test is suitable for each population. As mentioned in previous reviews, it is unlikely that there is one perfect screening instrument that can be used in every population and for all types of neurodegenerative and cerebrovascular brain diseases [9]. It is logical that the preferred characteristics for a cognitive screening test vary among settings. For example, a team of researchers that wants to exclude AD in their study or clinicians in a first aid setting probably prefer a very quick and easy-to-interpret cognitive screening test whereas in a memory clinic a somewhat longer test with the inclusion of different cognitive domains could be chosen. Therefore, there is a need for a new up-to-date overview of all currently available screening instruments and their psychometric properties for the detection of AD. This systematic review will take into account different screening settings.

## Method

This review consists of two parts. First, a systematic search for relevant screening instruments was performed. Second, a search was carried out to identify the psychometric properties of these screening instruments.

### Search strategy to identify screening instruments

In order to identify relevant publications about screening instruments, a systematic literature search was conducted. The following electronic databases were used: Web of Science and MEDLINE. Together, these databases provide a broad coverage of (neuro)psychological and medical journals published worldwide.

The literature search was conducted in February and March 2015, updated in April 2016 and May 2018. Combinations of the search terms “cognitive screen*” and “mild cognitive impairment” and/or “*Alzheimer*” were used. In addition, the search terms “screen*” and “computer” in combination with “mild cognitive impairment” and/or “*Alzheimer*” was used to identify computer screening instruments. We limited the search to studies published after 1991 in English or Dutch. Additionally, the bibliographies of published studies, particularly previous systematic reviews, were also used to search for potentially relevant screening instruments.

**Fig. 1:**
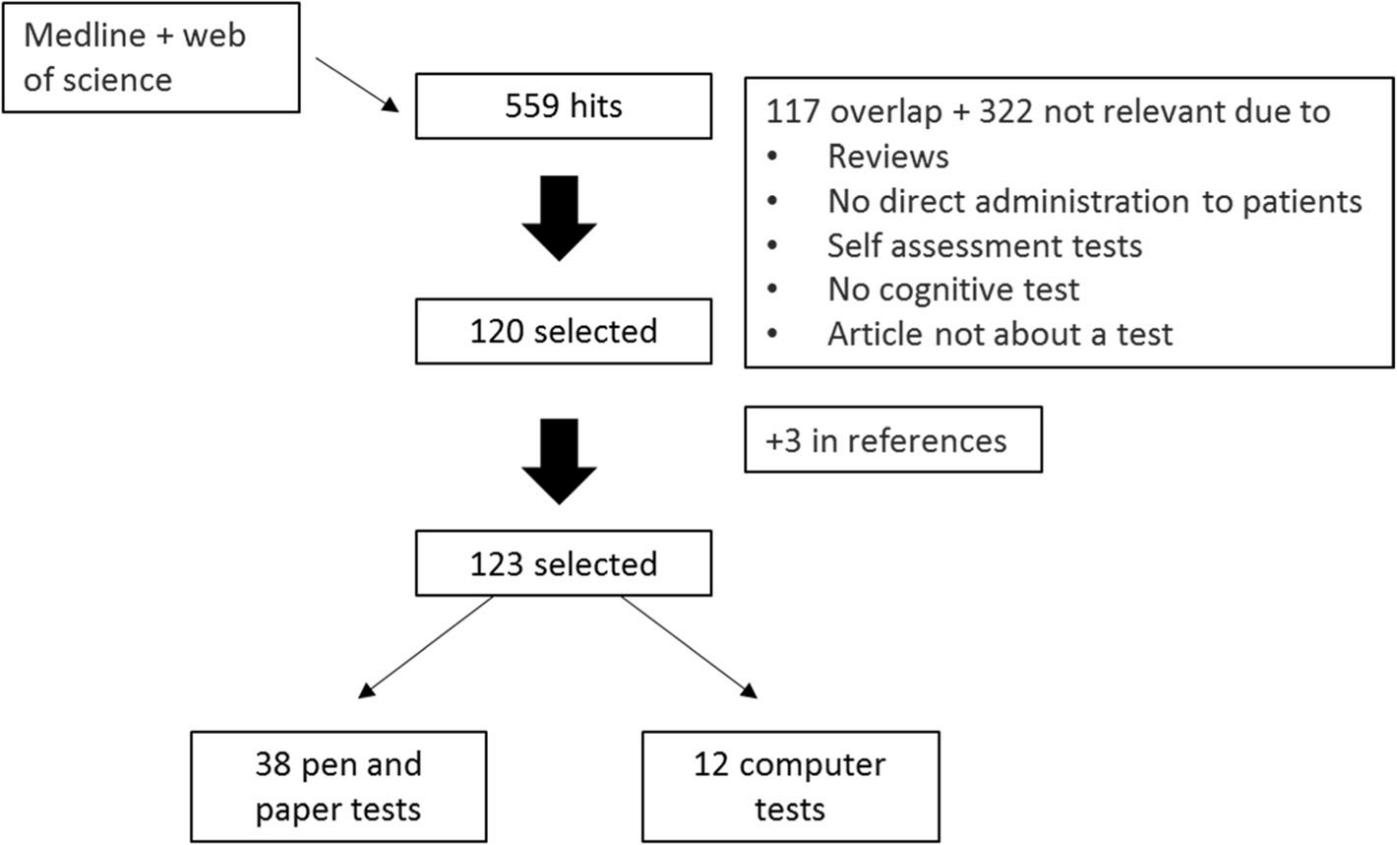
Search strategy to identify screening instruments

After reviewing the references, we selected the studies that described screening instruments with the following characteristics: (a) the test was designed to screen for cognitive impairment or could be used for that purpose, (b) the duration of the test was 20 min or less, (c) the test was available in English or Dutch, (d) the test was administered directly to patients (no informant-rated tests were used), (e) the test was not a telephone test, (f) the test was not a self-administration test, and (g) specifically for computer tests, an administrator was physically available (so no internet-based tools were included). The search resulted in 559 hits, 371 from the initial search, 127 from the first update, and 61 from the second. Thereof, 123 studies were selected that dealt with 38 different pencil and paper tests and 12 computer tests (Fig. 1). When all eligible studies about the screening instruments were identified, the relevant test variables and characteristics were extracted from the studies and summarized in a table (Tables 1, 2, and 3) (Additional file 1).

**Table 1:** Overview of the available pencil and paper tests (part 1: short screening instruments)

Tests between 2 and 5 min									
Abbreviation	Free?*	Duration in minutes	Memory	Language	Orientation	Executive functions	Praxis	Visuospatial abilities	Attention
CDT [15]	Yes	2				v		v	
AQT [24]	No	3–5							v
Qmci [25]	Yes	3–5			v	v		v	
Mini-Cog [26]	Yes	2–4	v					v	
Phototest [27]	Yes	3	v	v					
SPMT [28]	?	2–4	v						
MIS [29]	Yes	4	v						
RCS [30]	Yes	< 3	v			v		v	
6 CIT [31]	?	2–3	v		v				v
SIS [32]	Yes	2–5	v		v				
10-CS [33]	Yes	3	v	v	v				
K-D test [34]	No	1–2							v

*Free of charge

**Table 2:** Overview of the available pencil and paper tests (part 2: longer screening instruments)

Tests between 5 and 20 min									
Abbreviation	Free?*	Duration in minutes	Memory	Language	Orientation	Executive functions	Praxis	Visuospatial abilities	Attention
MMSE [5]	No	5–10	v	v	v		v	v	v
7 MS [35]	No	7–15	v		v	v			
ACE-R [36]	Yes	12–20	v	v	v			v	v
ACE-III [37]	Yes		v	v				v	v
M-ACE [38]	Yes	< ACE-R	v	v	v	v		v	
BCAT [39]	No	10–15	v	v	v	v		v	v
Brief KSCAr [40]	Yes	15	v		v	v		v	v
Mini-KSCAr [41]	Yes	10	v		v	v		v	
COST [42]	Yes	5–10	v	v	v	v	v	v	v
CONCOG [43]	Yes	5–10	v	v	v	v		v	
DemTect [44]	Yes	8–10	v	v					v
Eurotest [45]	Yes	8–9	v	v					v
FOME [46]	No	15	v						

*Free of charge

**Table 3:** Overview of the available pencil and paper tests (part 3: longer screening instruments)

Abbreviation	Free?*	Duration in minutes	Memory	Language	Orientation	Executive functions	Praxis	Visuospatial abilities	Attention
MES [23]	Yes	7	v			v			
MoCA [47]	Yes	10–15	v	v	v	v	v	v	v
MoCA-B [19]	Yes	15–21	v	v	v	v	v	v	v
SF-MoCA [48]	Yes	< MoCA	v		v				v
NUCOG [49]	No	15	v	v		v		v	v
R-QCST [50]	Yes	10–15	v	v		v		v	v
RUDAS [51]	No	20	v	v		v	v	v	
SCEB [52]	?	6–12	v	v	v	v		v	
SKT [53]	Yes	10–15	v						v
SLUMS [54]	Yes	10–15	v		v	v			v
STMS [55]	Yes	5–10	v		v	v	v	v	v
TE4D-cog [56]	Yes	10	v	v	v				v
LaSSI-L [57]	?	20	v						

*Free of charge

### Search strategy to identify the psychometric properties of the screening instruments

**Fig. 2:**
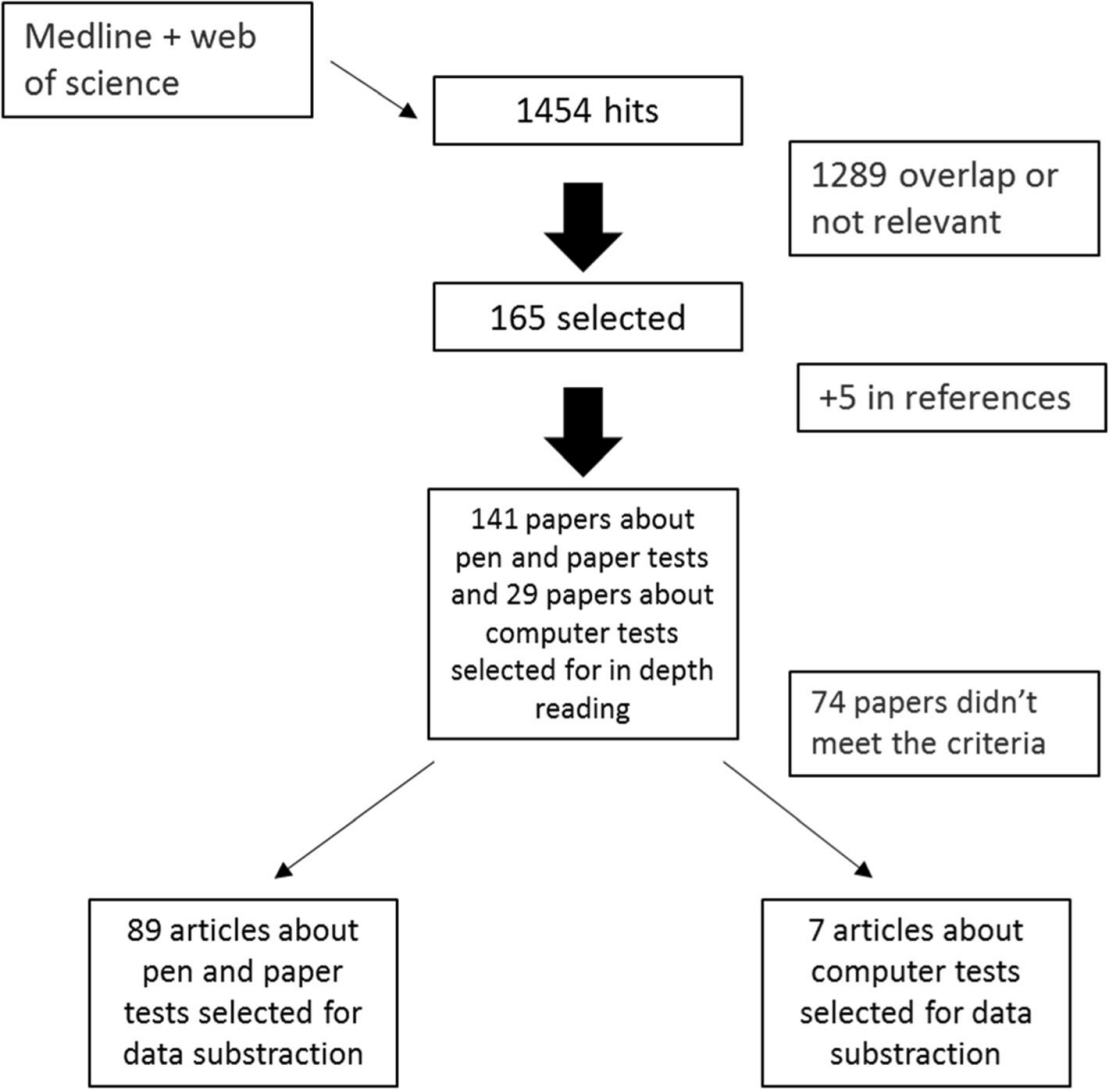
Search strategy for the identification of the psychometric properties of the screening instruments

Between March and July 2015 and during an update in April 2016 and May 2018, the same databases were searched again combining the names of each screening instrument with the search terms “Mild Cognitive Impairment” and/or “*Alzheimer*”. Once more, the search was limited to papers published after 1991 and written in English or Dutch, and bibliographies of published papers were studied to identify additional relevant articles. After reviewing the individual papers, studies for data extraction and analysis were selected based on the following criteria: (a) the paper provided basic information about sample selection and demographics, (b) the diagnostic criteria for MCI and/or AD were clearly described and met the standard diagnostic criteria [1, 10–12], (c) the instrument was used to detect MCI or AD dementia, and (d) the psychometric properties (sensitivity (sn), specificity (sp), reliability) of the instrument were clearly reported. The initial search and the updates resulted in 1454 references respectively, of which 89 articles about pencil and paper tests and 7 about computer tests were selected for data subtraction (Fig. 2).

First, the studies for each instrument were categorized based on the target population that was used for the validation (memory clinic versus population-based). As a screening instrument developed and validated in a (memory) clinic setting is not always appropriate to use for population screening, a distinction was made between studies validated in the general population and those validated in a (memory) clinic setting. Second, data were separately gathered for the following conditions: MCI versus healthy controls and early AD dementia versus healthy controls. Third, to compare the different instruments for each condition, a weighted average for sn, sp, area under the curve (AUC), test-retest reliability, inter-rater reliability, and internal reliability was calculated, based on population sizes (*n*).

## Results

### Available screening instruments

Tables 1, 2, and 3 show the details and characteristics of the 38 pencil and paper tests considered for further evaluation. In general, the tests can be divided in two groups. The first group contains 12 instruments that require 5 min or less to complete. Of these 12 instruments, four measure only one cognitive domain, while four instruments measure two cognitive domains and another four instruments measure three different cognitive domains. The Scenery Picture Memory Test (SPMT) and Memory Impairment Screen (MIS) only measure episodic memory. The Alzheimer Quick Test (AQT) only measures attention. Eight of these instruments include a memory task. As memory function is one of the first domains affected by AD pathology [13, 14], it could be seen as a limitation for AD screening that the clock drawing test (CDT), Alzheimer Quick Test (AQT), and Quick Mild Cognitive Impairment screen (Qmci) do not measure memory functioning.

The second group contains 26 tests that require an administration time between 5 and 21 min. All 26 tests measure memory functions. The Fuld Object-Memory Evaluation (FOME) and the Loewenstein-Acevedo Scale for Semantic Interference and Learning (LASSI-L) only measure memory function. All other 24 instruments measure in addition to memory functions one or more other domains. The Cognitive State Test (COST), the Montreal Cognitive Assessment (MoCA), and the MoCA-basic (MoCA-B) cover all seven cognitive domains described in Tables 1, 2, and 3, namely, memory, language, orientation, executive function, praxis, visuospatial abilities, and attention. The Mini-Mental State Examination (MMSE), the Brief Cognitive Assessment Tool (BCAT), and a Short Test of Mental Status (STMS) measure six of these cognitive domains. Eight of the 24 instruments measure five cognitive domains, four instruments measure four domains, three measure three domains, and only the Short Cognitive Performance Test (SKT) measures two domains. As mentioned above, the FOME is the only instrument that measures one domain.

Table 4 provides an overview of the 12 computer tests that were included in this study. Only one computer test (Inoue) requires less than 5 min to complete, while all other tests require above 7 min. All tests measure memory functioning, and except for the CANTAB-PAL and CANTAB mobile, they all measure multiple additional domains.

**Table 4:** Overview of the available computer tests

Abbreviation	Free?*	Duration in minutes	Memory	Language	Orientation	Executive functions	Praxis	Visuospatial abilities	Attention
Cogstate Brief Battery [58]	Yes	12–15	v			v			v
CANTAB mobile [59]	No	10	v						
CANTAB-PAL [60]	No	8–10	v						
MCI screen [61]	No	10	v	v		v			
CANS-MCI [62]	No	15–30	v	v		v			
CAMCI [63]	No	20–25	v			v			v
DETECT [64]	?	7–10	v			v			v
Computer test Kluger [65]	?	12–15	v		v	v	v		
CST/COGselftest [66]	?	15	v		v	v		v	v
Computer test Inoue [67]	?	4	v		v			v	
NCGG-FAT [68]	?	20–30	v			v		v	v
MoCA computer tool [69]	Yes	10–15	v	v	v	v		v	v

*Free of charge

### Psychometric properties of the screening instruments

For further evaluation, two tests were excluded. The first was the CDT, because it is part of several other tests such as the MoCA and it has no uniform scoring system. Moreover, Ehreke and colleagues concluded in their systematic review that due to its poor psychometric properties, the CDT should not be used for MCI screening [15]. We also excluded the CDT as a screen for AD as different scoring systems for the CDT make it difficult to compare different studies that used the CDT as a screen for AD. The second excluded test was the MMSE, because it has already been reviewed extensively by Mitchell who concluded that the MMSE is not appropriate to detect mild cognitive impairment [6].

#### Pencil and paper test: detection of mild cognitive impairment

Tables 5 and 6 summarize the different instruments and their psychometric properties validated in a (memory) clinic setting. From the 12 short-duration instruments, only seven were validated in a (memory) clinic setting for MCI. For five of these six instruments, there is only one study published; for the phototest and Qmci, there are two different studies published. Both the six-item screener (SIS) and the 10-point cognitive screener (10-CS) present with high sp for MCI but low sn. Based on sn, sp, and AUC, the Qmci and phototest score well, whereby the Qmci results might be more valid as it has been tested in more participants.

**Table 5:** Overview of the psychometric properties for pencil and paper tests validated in a (memory) clinic for MCI (part 1: short screening instruments)

Instrument	Number of articles*	*N* healthy controls	*N* MCI	*n*	Sensitivity	Specificity	*n*	AUC	*n*	Internal consistency	*n*	Inter-rater reliability	*n*	Test-retest reliability (mean time between tests)
Phototest	2	85	98	183	77.1	91.5	91	93						
Qmci	3	851	305	1416	85.7	85.7	307	92.3					30	0.93 (31.1 days)
AQT	1	47	75	122	80	76	122	85					122	0.88 (16 weeks)
SIS	1	375	440	815	34.3	90.1	815	63.2						
6 CIT	1	130	67	197	66	70	197	71						
10-CS	1	106	56	162	60.5	94.3	162	85						
K-D test	1	135	39	174	92.3		174	71						

*When more than 1 study is available, the weighted mean is calculated for the sensitivity, specificity, AUC, internal consistency, inter-rater reliability, and test-retest reliability

**Table 6:** Overview of the psychometric properties for pencil and paper tests validated in a (memory) clinic for MCI (part 2: longer screening instruments)

Instrument	Number of articles*	*N* healthy controls	*N* MCI	*n*	Sensitivity	Specificity	*n*	AUC	*n*	Internal consistency	*n*	Inter-rater reliability	*n*	Test-retest reliability (mean time between tests)
NUCOG	1	60	8	68	83.3	87.5			68	0.92				
MoCA	20	1628	1149	2777	83.9	74.6	2179	85.1	1801	0.78	724	0.97	1189	0.88 (3–24 weeks)
MoCA-B	1	43	42	85	81	86	85	90	85	0.82			25	0.91 (2 months)
SF-MoCA	1	28	43	71			71	86						
ACE-M	1	72	39	111	77	82								
ACE-R	7	334	276	610	82.8	77.4	546	87.1	528	0.87	522	0.94	430	0.95 (4–6 weeks)
Mini-KSCAr	1	21	27	48	81	95								
FBMS	1	80	23	103	82.6	87.5							103	0.65 (9 weeks)
SLUMS	2	491	237	1112	84.5	75.3	728	90.5	274	0.85				
SKT	1	56	82	138	100	84.8	138	99.1	138	0.8				
QCST	1	186	121	307	87.6	83.3	307	92.3					307	0.93 (32.1 days)
DemTect	3	182	195	516	84.1	91.5	153	96.8			242	0.99		
SCEB	1	48	27	75	75	86	75	86						
STMS	2	839	261	1100	68	76	1100	81.6						
MES	1	197	310	507	83.7	87.1	507	92.2			507	0.92		
RUDAS	2	228	82	88	56.8	90.3	228	79.7	77	0.71	77	0.88	77	0.90 (4 weeks)
RCS	1	33	61	94	87	70								
LASSI-L	1	44	15	59	73,3	93.2								

*When more than 1 study is available, the weighted mean is calculated for the sensitivity, specificity, AUC, internal consistency, inter-rater reliability, and test-retest reliability

For 18 of the 26 long-duration screening instruments, psychometric properties to screen for MCI are available. The MoCA is the best studied instrument, as it is covered in 20 papers, followed by the Addenbrooke’s Cognitive Examination Revised (ACE-R), described in seven studies. For both the ACE-R and the MoCA, sn is high (> 80%) while sp is rather low (respectively 74.6% and 77.4%). Based on the combination of sn (> 80%), sp (> 80%), and AUC (> 90%), the SKT, DEMTECT, Memory and Executive Screening (MES), Quick Cognitive Screening Test (QCST), and MoCA-Basic (MoCA-B) are promising tests to screen for MCI in a memory clinic. However, more studies are needed to confirm these results.

As shown in Table 7, only two instruments are validated in a population-based cohort, namely the MoCA and BCAT. Again the MoCA is with six studies the best studied instrument. For both tests, the psychometric properties are reasonable (sn and sp > 80%).

**Table 7:** Overview of the psychometric properties for pencil and paper tests validated in a population-based cohort for MCI

Instrument	Number of articles*	*N* healthy controls	*N* MCI	*n*	Sensitivity	Specificity	*n*	AUC	*n*	Internal consistency	*n*	Inter-rater reliability	*n*	Test-retest reliability (mean time between tests)
BCAT	1	49	26	75	81	80	75	90						
MoCA	7	11,738	4365	16,103	82.6	85.6	15,919	89.1	15,595	0.89			840	0.92 (6–35 days)

*When more than 1 study is available, the weighted mean is calculated for the sensitivity, specificity, AUC, internal consistency, inter-rater reliability, and test-retest reliability

#### Pencil and paper tests: detection of Alzheimer’s disease

The variables in Tables 8 and 9 are the same as those in Tables 5 and 6, but the instruments and their psychometric properties are validated in AD dementia instead of MCI. From the initial 11 short-duration instruments that were found, only six were validated for AD in a (memory) clinic setting. If we compare sn, sp, and AUC of the instruments with those of the validation in MCI (Tables 5 and 6), the values are higher. Based on the three statistical measures, the phototest seems to be the most accurate short-duration instrument, followed by the AQT and SPMT. The test-retest results of the AQT, MIS, and SPMT are good. The only instrument of which the inter-rater reliability was described is the SPMT, with good reliability. For the Mini-Cog, only results about sn were available.

**Table 8:** Overview of the psychometric properties for pencil and paper tests validated in a (memory) clinic for AD dementia (part 1)

Name	Number of articles	Healthy controls	AD	*n*	Sensitivity	Specificity	*n*	AUC	*n*	Internal consistency	*n*	Inter-rater	*n*	Test-retest (mean time between tests)
AQT	1	47	180	227	88	82	227	95					227	0.88 (16 weeks)
MIS	1	206	187	393	97								187	0.91 (?)
SIS	1	475	1061	1536	88.5	78.3	1536	83.8						
Phototest	1	30	56	86	89.3	96.7	86	97						
SPMT	1	54	128	182	84.8	89.8	182	92.5			128	0.750* 0.795**	128	0.898* 0.929** (2.3 months)
Mini-Cog	1	64	215	279	90.7									
K-D test	1	135	32	167	93.8		167	74						

*Pic 1, **pic 2

**Table 9:** Overview of the psychometric properties for pencil and paper tests validated in a (memory) clinic for AD dementia (part 2)

Name	Number of articles*	Healthy controls	AD	*n*	Sensitivity	Specificity	*n*	AUC	*n*	Internal consistency	*n*	Inter-rater	*n*	Test-retest (mean time between tests)
NOCUG	1	60	17	77	100	100								
MoCA	15	1552	1290	2842	92.7	91.4	1968	96.7	1715	0.84	562	0.98	874	0.86 (between 1 and 3 months)
ACE-R	4	299	254	553	96.3	82.7	477	98.1	255	0.89	255	1	45	0.91 (4 weeks)
7 MS	4	373	320	693	93.7	92.9	419	97.5	154	0.79	269	0.96	50	0.91 (1 to 2 months)
Mini-KSCAr	2	21	37	58	100	95					160	0.99		
FBMS	1	80	25	105	100	87.5	105	98.5					105	0.65 (9 weeks)
TE4D	1	25	178	203	100				203	0.87	203	1		
SKT	1	56	46	102	85.3	51.9	102	75.1						
RUDAS	1	23	23	46	100	74								
COST	1	114	74	188	81	99	188	94	114	0.88				
SCEB	2	95	78	173	93.4	88.5	173	96.1						
STMS	1	788	235	1023			1023	97						
MES	1	197	228	425	99	98.8	425	99.8			228	0.92		
Demtect	3	182	248	430	95.8	86.1	164	86.2			266	0.99		
SF-MoCA	1	28	20	48			48	93						
LASSI-L	1	44	19	63	78.9	97.7								

*When more than 1 study is available, the weighted mean is calculated for the sensitivity, specificity, AUC, internal consistency, inter-rater reliability, and test-retest reliability

Seventeen out of the 26 long-duration screening instruments are validated for AD. Again, the best studied instrument, described in 13 papers, was the MoCA, followed by the ACE-R and 7 MS, both described in four studies. The sn, sp, and AUC of both the MoCA and the MES were good (> 90%), making these the two superior tests. However, the psychometric properties of the other tests were, except for the SKT, all reasonable (> 74%). The sp of the SKT was inadequate (51.9%).

Table 10 demonstrates again that only a few screening instruments are validated in a population-based cohort. For the MoCA, two studies are available, while for the MIS, only one study is published. The sn of the MoCA is high (96.6%) with a reasonable sp (81.8%). The psychometric properties for the MIS, a very short instrument, are also good (all > 85%).

**Table 10:** Overview of the psychometric properties for pencil and paper tests validated in a population-based cohort for AD

Instrument	Number of articles*	*N* healthy controls	*N* AD	*n*	Sensitivity	Specificity	*n*	AUC	*n*	Internal consistency	*n*	Inter-rater reliability	*n*	Test-retest reliability (mean time between tests)
MIS	1	212	28	240	86.0	97.0	240	93						
MoCA	2	6357	313	6670	96.6	81.83			6576	0.85			35	0.96 (1 month)

*When more than 1 study is available, the weighted mean is calculated for the sensitivity, specificity, AUC, internal consistency, inter-rater reliability, and test-retest reliability

#### Computer tests

Table 11 gives an overview of the computer instruments that are validated in a clinic setting. The first part is the results for the instruments that are validated for MCI, and the second part contains the results for AD. As can be seen in the tables, most studies are underpowered as very few participants are included in the validation studies. Together, the studies evaluating the Cogstate include most participants. For the detection of MCI, the Cogstate and MoCA-CC have good psychometric properties. For the detection of AD, the CANS-MCI and Cogstate are preferred.

**Table 11:** Overview of the psychometric properties for computer tests

Instrument	Number of articles*	*N* healthy controls	*N* MCI/AD	*n*	Sensitivity	Specificity	*n*	AUC	*n*	Internal consistency	*n*	Inter-rater reliability	*n*	Test-retest reliability (mean time between tests)
Test validated for MCI in a (memory) clinic														
Cogstate	1	653	107	760	80.4	84.7	760	91.0						
CANTAB-PAL	1	22	17	39			39	80.3						
CANS-MCI	2	61	50	111	83.5	73.0	111	82.1	97	0.77			97	0.88 (3 months)
MoCA computer tool (MoCA-CC)	1	85	96	781	95.8	87.1	781	97.0						0.82 (6 weeks)
Test validated for MCI in a population-based cohort														
CAMCI 1		296	228	524	86	94.0			524	0.72				
Tests validated for AD in a (memory) clinic														
Cogstate	1	653	44	697	100.0	84.7	653	99.0					697	0.70 (4 months)
CANTAB-PAL	1	16	34	50	68.0	98.0								
CANS-MCI	1	41	21	62	100.0	97.0	62	98.0	97	0.77			97	0.88 (3 months)
Test Inoue	1	102	72	174	96.0	86.0								

*When more than 1 study is available, the weighted mean is calculated for the sensitivity, specificity, AUC, internal consistency, inter-rater reliability, and test-retest reliability

There is only one computer test validated in a population-based cohort, namely the Computer Assessment of Mild Cognitive Impairment (CAMCI) with a sn of 86% and a sp of 94%.

## Discussion

The aims of this review were to identify and evaluate available screening instruments for early detection of AD and MCI. As mentioned in the introduction, not for every setting the same tests are appropriate. Therefore, we will discuss briefly two general findings and subsequently discuss the applicability of the different cognitive screens in different settings.

### General findings

*A first general finding* is that a large number of screening instruments are available. However, for 10 out of the 38 instruments, no paper was found that clearly described the validation in MCI or AD. In addition, most instruments had only one paper dedicated to their validation. Besides, as Tables 5, 6, 7, 8, 9, and 10 show, only a limited amount of researchers reported reliability statistics for their instruments. Adequate reliability is essential for robust validity and should therefore be evaluated when validating an instrument. Another remark is the small sample sizes of some studies. Such underpowered studies may lead to misrepresenting results. In order to get representative results, studies with more participants are needed.

*A second general finding* is the paucity of studies describing the validation of computer instruments. Next to that, none of the reviewed computer instruments showed compelling evidence of superiority above pencil and paper tests despite some advantages of computerized tests, such as exact recording, highly standardized format, the possibility to adapt instructions or tasks to the abilities of the participants to avoid floor and ceiling effects and the convenience of automatic calculation of scores. The lack of studies describing the validation of computer instruments may be partly due to the negative association between older adults and computers. Indeed, a majority of older adults lack familiarity with computers, which can negatively affect performance [16, 17]. For example, according to a study of Perla and colleagues, participants that were unfamiliar with computers, female participants, and participants with a lower socioeconomic status were less inclined to do a computer screening for dementia [18]. Another explanation could be that we restricted our search to instruments that need an administrator, so web-based screening tools that can be administered on the home computer without the presence of a specialized administrator were excluded.

### Population screening

Population-based validation of screening tests is important, as it may (a) result in norms which can serve as reference values to evaluate how well an individual performs compared with the general population and (b) let us gain insight in normal aging. Moreover, good diagnostic instruments that can be used for population screening should be available when disease-modifying treatment options for AD become available [4]. However, the most important finding for population-based research is the lack of instruments that are validated in a population-based cohort. In this review, there is only one computer test, one short pencil and paper test, and two longer pencil and paper tests that are validated in a population-based cohort. The CAMCI is the only computer test that is validated for MCI in a population-based cohort. For AD, there were none. The CAMCI is an interesting instrument as its psychometric properties are good and it uses different tasks that are affected by AD, like navigation and memory.

The only short-duration instrument validated for AD dementia in a population-based cohort was the MIS. It showed good sn and sp to differentiate AD dementia patients from healthy controls. However, it is not validated for MCI. An explanation for the low number of short-duration instruments that was validated could be that they are often part of a broader neuropsychological examination. Therefore, they are not discussed as a screening instrument.

The psychometric properties of the MoCA, a longer duration instrument, are of all the population-based validated screens the best studied. Although the MoCA’s psychometric results score good, our results are somewhat less positive than those reported in a review of Julayanont and colleagues [19]. It is important to mention that Julayanont did not differentiate between a population-based or a (memory) clinic setting. In their review, sn for MCI detection was on average 86%, while the weighted average for the general population in our review is 82.6%. The sn in Julayanont’s review to detect AD was on average 97%, while the weighted average in this review is 96.6%. The sp for both MCI and AD together was on average 88%. In our study, the weighted average sp for MCI and AD is respectively 85.6% and 81.8% in a population-based cohort. The lower average sn and sp can be explained by the use of weighted averages in our review and by the fact that new studies were taken into account.

### Clinical setting

#### The short-duration instruments (< 5 min)

In some clinical settings (especially primary care), a short instrument is needed. This instrument’s purpose is to help to select older adults in need for a more detailed cognitive evaluation. The most crucial characteristic of these instruments is a short administration time. Therefore, we will below discuss all instruments with an administration time of 5 min or less.

For detection of MCI, the Qmci and phototest are preferable, with the Qmci being the most studied instrument. The Qmci is a modified version of the AB Cognitive Screen 135 (ABCS 135; [20]) which emphasizes the subtest from the ABCS 135 that best discriminated MCI from healthy controls, namely delayed recall and verbal fluency. The phototest assesses visual naming, verbal fluency, and episodic memory. The phototest is developed to be used in people with low education. Of the short-duration instruments, the SIS scored the weakest. Both SIS and 10-CS had high sp for MCI but low sn, indicating that the tasks are too easy for MCI patients (ceiling effect) and a lot of them are classified as false negatives. To detect AD, based on the statistical measures, the phototest is the best short-duration instrument, closely followed by the AQT, SPMT, and MIS. Again, the SIS scored low. Our results are somewhat in line with previous reviews. For example, Brodaty and colleagues concluded that the best very short screening instruments for a GP are the General Practitioner Assessment of Cognition (GPCOG), Mini-Cog, or the Memory Impairment Screen (MIS) [21]. Also, in a review of Lin and colleagues, the MIS was considered as a good instrument to detect cognitive impairment in older adults [22]. However, both reviews only took into account cognitive impairment in general and not specifically the early stages of impairment such as MCI due to AD.

Therefore, at this moment, we recommend to use the Qmci to detect MCI, the phototest to detect MCI in patients with low education levels, and the AQT, SPMT, or MIS to detect AD.

#### Long-duration screening instruments

For more specialized settings like for example a memory clinic or clinical trials, somewhat longer screening instruments are preferred. In this setting, the instruments are not solely used to screen for cognitive impairment, but can also be used for follow-up and for clinical trials to assess the patient’s response to treatment. Therefore, we will below discuss the instruments with an administration time between 6 and 20 min.

The results of our data search indicate that none of the long-duration screening instruments are suitable for detecting MCI. Especially, the balance between sp and sn forms an issue for a lot of instruments. For the ACE-M (sn 77.0%, sp 82.0%), RUDAS (sn 56.8%, sp 90.3%), and SCEB (sn 75.0%, sp 86.0%), the sn is relatively low, indicating that these instruments will allocate many MCIs as healthy controls. In contrast, the sp of the MoCA (sn 83.9%, sp 74.6%), ACE-R (sn 82.8%, sp 77.4%), RCS (sn 87%, sp 70.0%), and SLUMS (sn 84.5%, sp 75.3%) is rather low. With low sp, many healthy subjects will be classified as having MCI and be referred for further examination. This may elevate health care costs and worry the participants. Of all the instruments, the NUCOG, ACE-R, mini-KSCAR, SKT, Demtect, QCST, and MoCA-B have average psychometric results. The MoCA, however, is the best studied instrument, followed by the ACR-R. An advantage of the ACE-R is the availability of differential diagnosis profiles. For each individual, a profile is constructed that indicates the likelihood that their impairment is due to AD versus frontotemporal dementia. At the moment, the ACE-III, a new version of the ACE-R, is on the market. However, its value and specific statistical properties are not yet studied as much as the ACE-R, so whether the new version is better than its previous cannot be determined yet. For the detection of AD dementia, the MoCA and Memory and Executive Screening (MES) test perform best. The MES test is developed by Guo and colleagues for the detection of MCI and mild AD dementia and focuses on memory and executive functioning [23].

*It is worth mentioning* that all long-duration screening instruments measure episodic memory. This is positive and also logical as one of the first noticeable symptoms of AD are problems with episodic memory. Another important finding is that the MoCA is extensively studied. Interestingly, researchers are adapting the MoCA for different populations (for example, the MoCA-B for people with low education levels) and devices (for example, the MoCA-CC for the computer).

*From all the computer instruments*, the MoCA-CC (all statistics above 87%) and Cogstate (all statistics above sn, sp, and AUC ≥ 80%) show promising psychometric properties when used to detect MCI in a (memory) clinic setting. For the detection of AD dementia, the Cogstate, CANS-MCI, and computer test of Inoue all perform well. From these three tests, CANS-MCI is specifically designed to be sensitive for MCI and AD pathology, as it focuses on the cognitive domains affected by AD.

### Strengths and limitations

It should be mentioned that this review has its strengths and limitations.

A first limitation is that in our attempt to present as many instruments as possible, it was not feasible to conduct a detailed quality rating of each individual study from which we extracted the data presented in this review. However, by applying strict inclusion criteria, for example by excluding studies using wrong diagnostic criteria, for example identifying MCI or AD with a screening instrument, for AD, MCI, and healthy controls, this limitation was minimized as much as possible.

Another limitation is the lack of information, provided by authors of the selected studies, about the power of the instrument to differentiate between different forms of dementia. With most instruments, it is therefore not possible to differentiate between participants with probable AD or other causes of dementia such as Lewy body dementia or vascular dementia. Almost all screening instruments use a simple dichotomized cutoff (cognitively impaired or not), while it could be more interesting for clinicians to have a short instrument that distinguishes between different etiologies. However, the measurement of different cognitive domains is needed, to make differential profiles which would increase the administration time. Especially for very short instruments, the ability to differentiate between dementias may be questionable. For longer screening tests such as the ACE-R, this is already possible, but it is time-consuming for the administrator. Hence, differential diagnostic power may also be beneficial for computer instruments, particularly if the profiles are automatically calculated.

It can be seen as a shortcoming that we did not provide information about the instrument’s ability to differentiate between MCI and AD dementia. However, in our opinion, the aim of a screening instrument is to identify cognitive impairment or, if screening for AD, cognitive impairment that points in the direction of AD pathology. In both cases, further research is needed. In clinical practice, (instrumental) activities of daily living ((I)ADL) functioning is crucial to differentiate between the MCI and the dementia stage [1–3]. Hence, differentiating between MCI and AD with a cognitive screening instrument, without measuring (I)ADL functioning, would have little clinical benefit.

A next limitation is that we restricted our search to instruments that need an administrator and include only a measurement of the participant themselves. Due to this restriction, some promising screening instruments, such as the GPCOG, informant-rated questionnaires, or web-based screening tools, were not evaluated and discussed in this review.

A following limitation includes that we only discuss in this review a few psychometric properties. These are the psychometric properties that are most commonly described in the included studies. However, other psychometric properties such as construct and criterion or predictive validity were not discussed. A last limitation is that within this review, MCI is treated as one concept, despite the fact that most clinicians and researchers differentiate between different subtypes of MCI (amnestic, non-amnestic, multiple or single domain) [11]. However, the majority of the included studies grouped all MCI types as one diagnostic group or did analyses on the whole MCI group.

### Further research

More instruments should be tested in population-based cohorts. In addition, the balance between sp and sn should be improved through further research. To this end, it could be helpful to take into account the pathophysiological background of AD. Within this light, it may be interesting to improve the existing instruments and potentially adapt them for several settings and populations, such as the adaptation of the MoCA for lower educated people or for people with hearing impairments [19, 22]. Besides, the sp of the MoCA needs to be improved. Additionally, more research is needed for computer instruments, especially to adapt them for different devices (tablet, home computer), how they influence cognitive processes, and which device is most appropriate for older adults with and without computer experience. Finally, future reviews could focus on the screening ability of the instruments for the different subtypes of MCI and dementia and the ability of the instruments for other purposes such as measuring progression of cognitive decline and evaluating potential treatment effects and could include additional psychometric properties such as predictive values.

### Recommendations

Clinicians and researchers should abandon the idea that one screening instrument (like the MMSE) can be used in every setting, for all different neurodegenerative diseases and for each population. Tables 5, 6, and 12 summarize our recommendations. For the detection of MCI and AD in a population-based cohort, the MoCA is the most suitable instrument. If a shorter instrument is needed, the MIS can be used to detect AD dementia. In a (memory) clinic setting, the Qmci is a suitable short-duration screening instrument, while the phototest can be used for people with lower education levels The NUCOG, ACE-R, mini-KSCAR, SKT, Demtect, QCST, and MoCA-B are good candidates to choose from the longer screening instrument. The best studied test is the MoCA. If differential diagnosis is needed, the ACE-R is preferred. For the detection of AD dementia, the MoCA and MES can be used. For the detection of MCI and AD, the MoCA-CC and Cogstate seem promising computer instruments.

**Table 12:** Overview of the recommended tests

	Recommended tests to detect MCI	Recommended tests to detect AD
Population screening		
Short screening	/	MIS
Longer screening	MoCA CAMCI	MoCA
(Memory) clinic setting		
Short screening	Qmci Phototest (lower education)	MIS
Longer screening	MoCA/MoCA-CC ACE-R Mini-KSCAR NUCOG DEMTECT Cogstate	MoCA, MES

## Additional file

Additional file 1: Detailed overview of the studies included in this review. (DOCX 29 kb)

## Abbreviations

10-CS: 10-point cognitive screener
6 CIT: Six-Item Cognitive Impairment Test
7 MS: 7-min screening test
ABCS 135: AB Cognitive Screen
ACE-III: Addenbrooke’s Cognitive Examination III
ACE-R: Addenbrooke’s Cognitive Examination Revised
AD: Alzheimer’s disease
AQT: Alzheimer’s Quick Test
BCAT: Brief Cognitive Assessment Tool
Brief KSCAr: Brief Kingston Standardized Cognitive Assessment revised
CAMCI: Computer Assessment of Mild Cognitive Impairment
CANS-MCI: Computer-assisted Neuropsychological Screening for MCI
CANTAB mobile: Cambridge Neuropsychological Test Automated Battery Mobile
CANTAB-PAL: Cambridge Neuropsychological Test Automated Battery-Paired Associate Learning
CDT: Clock Drawing Test
CONCOG: Concise Cognitive Test
COST: Cognitive State Test
CST: The computerized self-test
FOME: Fuld Object-Memory Evaluation
K-L test: King-Devick test
LASSI-L: Loewenstein-Acevedo Scale for Semantic Interference and Learning
M-ACE: Mini Addenbrooke’s Cognitive Examination
MCI screen: Mild cognitive impairment screen
MCI: Mild cognitive impairment
MES: Memory and Executive Screening
Mini-KSCAr: Mini Kingston Standardized Cognitive Assessment revised
MIS: Memory Impairment Screen
MMSE: Mini-Mental State Examination
MoCA: Montreal Cognitive Assessment
MoCA-B: Montreal Cognitive Assessment Basic
NCGG-FAT: National Center for Geriatrics and Gerontology functional assessment tool
NUCOG: Neuropsychiatry Unit Cognitive Assessment Tool
Qmci: Quick Mild Cognitive Impairment screen
RCS: Rapid Cognitive Screen
R-QCST: Revised Quick Cognitive Screening Test
RUDAS: Rowland Universal Dementia Assessment Scale
SCEB: Short Cognitive Evaluation Battery
SF-MoCA: Short-form Montreal Cognitive Assessment
SIS: Six-item screener
SKT: Short Cognitive Performance Test (Syndrom Kurztest)
SLUMS: Saint Louis University Mental Status examination
sn: Sensitivity
sp: Specificity
SPMT: Scenery Picture Memory Test (SPMT)
STMS: Short Test of Mental Status
TE4D-cog: Cognitive tests of the Test for the Early Detection of Dementia from Depression
