# Supplementary material for: Brief cognitive screening instruments for early detection of Alzheimer’s disease: a systematic review
Source: Alzheimers Res Ther. 2019 Feb 28;11:21. doi: 10.1186/s13195-019-0474-3 (PMC6396539; doi:10.1186/s13195-019-0474-3)
Supplement: Supplementary file 1 — Detailed overview of the studies included in this review. (DOCX 29 kb) [file 13195_2019_474_MOESM1_ESM.docx]

| ***An overview of the studies included in table 3 part 1. short screening instruments*** | | |
| --- | --- | --- |
| **instrument** | **study** | **language** |
|  |  |  |
| **tests between 2-5 minutes** |  | |
| **phototest** | Carnero-Pardo et al. 2011 | Spanish |
|  | Russo et al. 2014 | Spanish (Argentina) |
| **Qmci** | Guo et al. 2010 | Chinese |
|  | O’Caoimh et al. 2012 | English |
|  | bunt et al . 2015 | Dutch |
| **AQT** | Takahhashi et al. 2011 | Japanese |
| **SIS** | Chen et al. 2010 | Chinese |
| **6 CIT** | Abdel-Aziz et al. 2015 | English |
| **10-CS** | Apolinario et al. 2016 | Portuguese (Brazil) |
| **K-D test** | Galetta et al. 2017 | English |

| ***An overview of the studies included in table 3 part 2 longer screening instruments*** | | |
| --- | --- | --- |
| **instrument** | **study** | **language** |
|  |  |  |
| **tests between 5-20 minutes** |  | |
|  |  |  |
| **NUCOG** | Barekatain et al. 2010 | Persian |
| **MoCA** | Goldstein | English |
|  | Thissen et al. 2010 | Dutch |
|  | Freitas et al.2011 | Portuguese |
|  | Julayanont | English |
|  | Smith et al. 2007 | English |
|  | Yeung et al. 2014 | Cantonese |
|  | Kaya et al. 2014 | Turkish |
|  | Roalf et al. 2012 | English |
|  | Hu et al. 2013 | Chinese (Beijing Version) |
|  | Magierska 2012 | Polish |
|  | Fujiwara et al. 2010 | Japanese |
|  | Nasreddine et al 2005 | English and French |
|  | Cummings-Vaughn et al 2014 | English |
|  | Lifshitz et al. 2012 | Hebrew |
|  | Gil et al. 2015 | Spanish |
|  | Chu te al. 2015 | Cantonese |
|  | Ng et al. 2013 | Singapore (different languages?) |
|  | Memoria et al. 2013 | Portuguese |
|  | Lee et al. 2008 | Korean |
|  | Janelidze et al. 2017 | Georgian |
| **MoCA-B** | Julayanont et al. 2015 | Thai |
| **SF-MoCA** | Larner et al. 2017 | English |
| **ACE-M** | Larner et al. 2015 | English |
| **ACE-R** | Mioshi et al, 2006 | English |
|  | Fang et al. 2013 | Chinese |
|  | Wong et al. 2013 | Chinese (Cantonese) |
|  | Alexopoulos et al. 2010 | German |
|  | Bastide et al. 2012 | French |
|  | Yoshida et al. 2012 | Japanese |
|  | Gonçalves et al. 2015 | Portuguese |
| **mini-KSCAr** | Heinik and Kavé 2015 | Hebrew |
| **FBMS** | Loewenstein et al.2009 | English |
| **SLUMS** | Tariq et al,2006 | English |
|  | Cummings-Vaughn et al 2014 | English |
| **SKT** | Flaks et al. 2009 | Portuguese |
| **QCST** | Guo et al. 2010 | Chinese |
| **DemTect** | Kalbe et al. 2004 | English |
|  | Scheurich et al. 2005 | German |
|  | Wojtynska, 2016 | Polish |
| **SCEB** | Girtler et al, 2012 | Italian |
| **STMS** | Tang-Wai et al. 2003 | English |
|  | Cummings-Vaughn et al 2014 | English |
| **MES** | Guo et al. 2012 | Chinese |
| **RUDAS** | Basic et al. 2009 | English |
|  | Chen et al. 2016 | Chinese |
| **RCS** | Malmstorm et al. 2015 | English |
| **LASSI-L** | Wesson et al. 2017 | English |

| ***An overview of the studies included in table 4*** | |  |
| --- | --- | --- |
| **instrument** | **study** | **language** |
|  |  |  |
| **BCAT** | MacDougall et al. 2015 | English |
| **MoCA** | tan et al. 2015 | Chinese |
|  | Lu et al. 2011 | Cantonese (Beijing version) |
|  | Rahman et al. 2009 | Arabic |
|  | Yu et al. 2012 | Beijing version |
|  | Dong et al. 2013 | English or Chinese dialects |
|  | Luis et al. 2009 | English |
|  | Zhou et al. 2014 | Chinese (Beijing Version?) |

| ***An overview of the studies included in table 5 part 1. short screening instruments*** | | |
| --- | --- | --- |
| **instrument** | **study** | **language** |
|  |  |  |
| **AQT** | Takahhashi et al. 2011 | Japanese |
| **MIS** | Chopard et al. 2007 | French |
| **SIS** | Chen et al. 2010 | Chinese |
| **Phototest** | Russo et al. 2014 | Spanish (Argentina) |
| **SPMT** | Takechi et al. 2010 | Japanese |
| **mini-Cog** | Milian et al 2012 | German |
| **K-D test** | Galetta et al. 2017 | English |

| ***An overview of the studies included in table 5 part 2. longer screening instruments*** | | |
| --- | --- | --- |
| **instrument** | **study** | **language** |
|  |  |  |
| **MoCA** | Freitas et al.2011 | Portuguese |
|  | Karunaratne | Sinhala |
|  | Memoria et al. 2013 | Portuguese |
|  | Yeung et al. 2014 | Cantonese |
|  | Kaya et al. 2014 | Turkish |
|  | Roalf et al. 2012 | English |
|  | Hu et al. 2013 | Chinese (Beijing Version) |
|  | Magierska 2012 | Polish |
|  | Fujiwara et al. 2010 | Japanese |
|  | Lee et al. 2008 | Korean |
|  | Nasreddine et al 2005 | English and French |
|  | Chang et al. 2012 | Mandarin |
|  | Wang et al. 2013 | Mandarin |
|  | chu te al. 2015 | Cantonese |
|  | Ng et al. 2013 | Singapore |
| **ACE-R** | Fang et al. 2013 | Chinese |
|  | Alexopoulos et al. 2010 | German |
|  | Yoshida et al. 2012 | Japanese |
|  | [Carvalho et al. 2010](http://www.ncbi.nlm.nih.gov/pubmed/?term=Carvalho%20VA%5BAuthor%5D&cauthor=true&cauthor_uid=20299857) | Portuguese |
| **7MS** | Solomon et al. 1998 | English |
|  | Meulen et al. 2003 | Dutch |
|  | Ijuin et al. 2008 | Japanese |
|  | Sungkarat et al. 2011 | Thai |
| **mini-KSCAr** | Hopkins et al. 2013 | English |
|  | Heinik and Kavé 2015 | Hebrew |
| **FBMS** | Loewenstein et al.2009 | English |
| **TE4D** | Mahoney et al. 2005 | English |
| **SKT** | Flaks et al. 2009 | Portuguese |
| **RUDAS** | Lype et al. 2006 | Malayalam |
| **COST** | Babacan-Yildiz et al. 2013 | Turkish |
| **SCEB** | Girtler et al, 2012 | Italian |
|  | Robert et al. 2003 | French |
| **STMS** | Tang-Wai et al. 2003 | English |
| **mes** | Guo et al. 2012 | Chinese |
| **Demtect** | Kalbe et al. 2004 | English |
|  | Scheurich et al. 2005 | German |
|  | Wojtyńska and Szcześniak, 2016 | Polish |
| **SF-MoCA** | Larner et al. 2017 | English |
| **LASSI-L** | Wesson et al. 2017 | English |

| ***An overview of the studies included in table 6*** | |  |
| --- | --- | --- |
| **instrument** | **study** | **language** |
|  |  |  |
| **MIS** | Kuslansky et al. 2002 | English |
| **MoCA** | Lu et al. 2011 | Cantonese (Beijing version) |
|  | Luis et al. 2009 | English |

| ***An overview of the studies included in table 7*** |  |  |
| --- | --- | --- |
| **instrument** | **study** | **language** |
|  |  |  |
|  |  |  |
| **Test validated for MCI in a (memory) clinic** |  |  |
| **Cogstate** | Maruff 2013 | English |
| **CANTAB-PAL** | Junkkila et al. 2012 | Finnish |
| **CANS-MCI** | Memória et al. 2014 | Portuguese |
|  | Ahmed et al. 2012 | English |
| **MoCA-Computer tool (MoCA-CC)** | Yu et al. 2015 | Chinese (Beijing) |
| **Test validated for MCI in a population based-cohort** |  |  |
| **CAMCI** | Saxton et al. 2009 |  |
| **Tests validated for AD in a (memory) clinic** |  |  |
| **Cogstate** | Maruff 2013 | English |
| **CANTAB-PAL** | O’Connell et al. 2004 | English |
| **CANS-MCI** | Memória et al. 2014 | Portuguese |
| **test Inoue** | Inoue et al. 2009 | Japanese |
